# Assessing Antimicrobial Efficacy on Plastics and Other Non-Porous Surfaces: A Closer Look at Studies Using the ISO 22196:2011 Standard

**DOI:** 10.3390/biology13010059

**Published:** 2024-01-20

**Authors:** Teresa Bento de Carvalho, Joana Bastos Barbosa, Paula Teixeira

**Affiliations:** Universidade Católica Portuguesa, Laboratório Associado, CBQF—Centro de Biotecnologia e Química Fina, Escola Superior de Biotecnologia, Rua Diogo Botelho 1327, 4169-005 Porto, Portugal; s-mtmcarvalho@ucp.pt (T.B.d.C.); pcteixeira@ucp.pt (P.T.)

**Keywords:** antimicrobial coating, high-touch surfaces, antimicrobial efficacy, pathogen transmission

## Abstract

**Simple Summary:**

Standardised antimicrobial testing methods are essential to validate the antimicrobial efficacy of materials and enable their application in real-life settings by providing reliable results that allow for comparison between antimicrobial surfaces while assuring end-use product safety. In this review, the literature on the ISO 22196:2011 protocols used in the published studies will be analysed.

**Abstract:**

The survival and spread of foodborne and nosocomial-associated bacteria through high-touch surfaces or contamination-prone sites, in either healthcare, domestic or food industry settings, are not always prevented by the employment of sanitary hygiene protocols. Antimicrobial surface coatings have emerged as a solution to eradicate pathogenic bacteria and prevent future infections and even outbreaks. Standardised antimicrobial testing methods play a crucial role in validating the effectiveness of these materials and enabling their application in real-life settings, providing reliable results that allow for comparison between antimicrobial surfaces while assuring end-use product safety. This review provides an insight into the studies using ISO 22196, which is considered the gold standard for antimicrobial surface coatings and examines the current state of the art in antimicrobial testing methods. It primarily focuses on identifying pitfalls and how even small variations in methods can lead to different results, affecting the assessment of the antimicrobial activity of a particular product.

## 1. Introduction

Antimicrobial materials have gained popularity over the last few years within the food and healthcare industries, due to their ability to safeguard food-contact surfaces, high-touch surfaces and medical devices by preventing microbial adherence and biofilm formation. Consequently, they help deter the spread of foodborne and nosocomial pathogens [1,2,3].

According to the latest report on zoonoses published by the European Food Safety Authority (EFSA) and the European Centre for Disease Prevention and Control (ECDC) in 2021, there were 4005 foodborne outbreaks across 27 member states of the European Union and the United Kingdom (Northern Ireland), resulting in 32,543 cases, including 2495 hospitalisations and 31 deaths [4].

The observed increase in foodborne outbreaks compared to the 2020 report is an important indicator that foodborne diseases are a growing challenge. Notably, more than one-third of these European outbreaks are associated with domestic settings [4], highlighting the need to prevent cross-contamination in such environments. Improper food handling and inadequate hygiene protocols elevate the risk of cross-contamination of surfaces or food products, threatening food safety assurance [5,6]. Concurrently, the European Centre for Disease Prevention and Control estimates that 4.5 million healthcare-associated infections occur in European hospitals yearly, as reported in a previous study [7], with more than half of these infections being preventable. The contamination of hospital surfaces or contaminated medical devices plays a significant role in the spread of pathogens and has been identified as the most likely transmission route [8]. These infections could be effectively prevented by using antimicrobial surface coatings (AMCs), which, while not a definitive solution, can help reduce the risk of infection by preventing viable bacteria from adhering to the surface and/or inhibiting their growth [9].

The mechanisms of action by which AMCs act on surfaces are classified into two categories: antimicrobial-releasing methods and contact-killing methods (i.e., potentiated surfaces and substances that do not allow for bacterial adhesion) [10]. Internationally recognised organisations provide standardised test methods to test the antimicrobial efficacy of AMCs, which fall into five categories that will vary according to the mechanism of action reported by the manufacturer: high surface-to-volume ratio tests such as ISO 22196 [11], adhesion tests such as ISO/TR 19402 [12], biofilm tests such as ISO/DIS 4768 [13], inhibition zone tests such as ISO 20776-2 [14] and suspension tests such as EN 1276 [15]. Cunliffe et al. [16] reported that modifications and iterations of the standardised protocol are common, either because of the preferences of the laboratory performing the test or because of protocol optimisation due to the microorganisms or the compound used. Any modification of a standard will affect the extrapolation of the results and cast doubt on its validity [16].

This review aims to critically assess the standardised ISO 22196 antimicrobial efficacy test [11], highlighting its potential weaknesses and emphasising the lack of evidence-based efficacy test protocols, which hinder the development and application of antimicrobial surface-coating technologies in clinical and industrial settings. 

## 2. Standardised Antimicrobial Efficacy Testing Method ISO 22196 (2011)

The standardised ISO 22196 antibacterial efficacy test method [11], regarded as the most widely used test method in the industry [11,16,17], delineates an in vitro approach for evaluating antibacterial activity on treated plastics and other non-porous surfaces. This method quantitatively assesses biocidal or bacteriostatic effects via direct contact between a liquid bacterial culture and control/test surfaces. Essentially, the protocol involves applying a known concentration and volume of *Staphylococcus aureus* or *Escherichia coli* inocula to the presumed antimicrobial surface, covering it with a plastic film, placing it in a Petri dish and then incubating the dish for 24 ± 1 h at 35 ± 1 °C and a relative humidity of not less than 90%. The recovery of bacteria from the test specimens is performed immediately after inoculation for the control surfaces and after 24 h of incubation for the control and test surfaces [11] by the addition of a neutralising solution. After recovering the bacteria from the test specimens, they are serially diluted and plated on nutrient agar. This standard has been proven reliable for testing the biocidal activity of active materials and surface coatings. However, it has also been demonstrated that it does not accurately represent real-life scenarios due to its artificial experimental conditions (i.e., temperature, incubation temperature and relative humidity). As a result, the extrapolation of results to real industrial or clinical settings can be challenging [1,18]. 

The pros of this antimicrobial efficacy test have been described as its simplicity, affordability and wide availability. It is also considered ideal for screening surfaces as a “proof of principle” test. This standard method is appropriate for antimicrobial-releasing coatings and contact-killing-based methods since the small volume of the all-in-one-plating method forces direct contact of the bacterial suspension with the test material [19]. Standardised tests play a pivotal role in developing novel antibacterial agents. It is necessary to implement standard methods that will echo safe end-use and real-life conditions in situ, with regulatory guidance being made available to researchers and commercial stakeholders interested in providing AMCs products to benefit public health [18,20]. This fact is crucial to understanding why AMCs have not been widely used in healthcare and community settings to date. The lack of studies conducted under real-life conditions to validate the effectiveness and benefits of this technology overshadows the risk of spreading multidrug-resistant (MDR) organisms [21].

## 3. Factors Affecting the Validity of the Antimicrobial Test Method ISO 22196 

A PubMed search for “ISO 22196” and “ISO22196” between January 2010 and October 2023 yielded 54 results for scientific reports, excluding 3 literature reviews that did not meet the selection criteria. This literature search showed that 48 scientific reports, comprising 88% of the studies that used the method, applied relevant modifications to the protocol; 3 entries did not provide information on the detailed protocol that was followed, as previously reported by Wiegand et al. [1]. Several authors reported that the currently used test protocol of choice for testing surfaces that made antimicrobial claims, ISO 22196, was performed with modifications to the original [22,23,24,25,26,27,28,29,30,31,32,33,34,35,36,37,38,39,40,41,42,43,44,45,46,47,48,49,50,51,52,53,54,55,56,57,58,59,60,61,62,63,64,65,66,67,68]. These modifications to the standard and the reported results of the literature search are shown in Table 1.

Most authors report that ISO 22196 [11], without modifications, fails to accurately simulate real environmental and usage conditions and allows for inflated values or erroneous claims of antimicrobial activity, thus compromising the validity of the efficacy result [17,29,37,68]. Since the parameters required by the standard are the optimum conditions to ensure the efficacy of the antibacterial activity of the surfaces, they will not produce the same efficacy results when applied in real-life environments such as hospitals, the food industry and household use, making this protocol a fictitious claim of antibacterial efficacy by the product. Modifications typically involve adjusting the incubation temperature, bacterial suspension volume and density and also using different culture media than those specified in the standard protocol. These are performed with the intent of replicating real-world use scenarios and are, thus, a tentative approach to recognising the true antimicrobial efficacy of the test product [1,16]. The results reported are not always obtained by considering the calculation formula established by the standard. Even though most studies report their results by giving the antibacterial activity (R) value, some solely report the number of viable bacteria (CFU) or log reduction values. As highlighted in Table 1, the reporting of the results varies greatly, with authors choosing a different expression of results than that requested by the standard (R). The reported methodology used to obtain the results is based on modified protocols and does not follow a clear expression of the results (as listed in Table 1).There is also a lack of distinction between the terms employed, namely, antibacterial efficacy/activity, bacteriostatic effects and bactericidal action.Hence, not allowing for the proper interpretation and comparison of the results when comparing the same microorganisms and antibacterial compounds, hindering and compromising their communication [1].

Many factors can influence the results of this testing method. Wiegand et al. [1] established a round-robin test, meaning an interlaboratory test performed independently several times, to evaluate the antimicrobial activity of biomaterials. This study states that four main factors influence the final result and, consequently, the resulting data and conclusions: (i) the incubation time of the Petri dishes, (ii) the initial level of the inoculum, (iii) the physiological state of the bacteria and (iv) the nutrient concentration of the nutrient source during incubation. It also showed that there was a wide variation in the results, depending on the laboratory to which the ISO 22196 test was attributed (seven in total) [1]. Humidity, contact time, airflow and surface topography also play a critical role in the result, as reported by Cunliffe et al. [16].

### 3.1. Inoculum

Low inoculum density has been associated with higher antimicrobial activity; bacteria may appear susceptible when using the standard inoculum (10^5^ CFU/mL) but appear resistant if the inoculum size is increased [69]. This relationship has been widely studied in the case of antibiotic susceptibility [70]. However, García et al. [70] have reported that at high inoculum levels, cells exhibit reduced absorption of disinfectants, yet the underlying mechanism behind this phenomenon remains relatively unknown and understudied [70]. From the literature reviewed, it appears that changes in the density of the test inoculum are not frequent, with only three authors [34,52,55] reporting the use of higher inoculum densities, namely, Barzan et al. [52], Richert et al., [55] and Yamada et al. [34], who opted for 1.0 × 10^6^ CFU/mL, 10^6^ CFU/mL and 0.4 to 3.0 × 10^8^ CFU/mL, respectively. These authors provided no comments on whether this change in densities significantly affected the antibacterial activity efficacy of the compounds tested. Conversely, 37% of the authors (20 studies) [2,8,24,27,31,32,38,39,40,41,46,47,50,52,54,56,59,66,68] reported an adjustment of the bacterial inoculum volume due to the size of the test specimen used. This modification has not been reported to influence the result of the protocol since ISO 22196 allows for different sizes/measurements of the test specimen and plastic cover film, as long as the inoculum volume is adjusted to be proportional to the area of the cover film used. The ISO 22196 protocol uses poor media (nutrient broth 1/500) to prepare the test inoculum, to ensure that the bacterial growth is not potentiated. Since antibacterial activity is impacted by shifts in the concentration of the nutrient broth used or by the use of richer media, as reported by Wiegand et al. [1], it is important to note that increasing the availability of nutrients in the media will result in lower antibacterial activity. Some authors [17,18,22,29,37,50,56] have reported changes in the media used to grow the bacterial inoculum. Ando et al. [22] have reported that NB 1/500 is a poor choice of media for evaluating the antibacterial activity of biomaterials since the bacteria inoculated in clinical biomaterials are killed by the lack of nutrients rather than by the active compound that needs to be tested.

### 3.2. Temperature and Humidity

Regarding temperature, low temperatures (<10 °C) can inhibit the antimicrobial activity of surface disinfectants, while high temperatures (>40 °C) can degrade and weaken the antimicrobial compound [71]. Temperature and airflow have a significant impact on the drying time of the inoculum, and ISO 22196 [11] does not mimic a real environmental condition due to the high temperature (35 ± 1 °C) and high relative humidity conditions (90%) [18]. This can lead to a slower drying time of the inoculum on the test surface, due to the high humidity under which the standard must be performed, thus enhancing the efficacy of the antimicrobial activity. Consequently, these exacerbated results will trigger unreliable and erroneous claims of antimicrobial efficacy [72]. Since humidity affects how long liquids take to dry, this is most likely related to the efficacy of AMCs. Reductions in humidity typically reduce antibacterial efficacy because evaporation reduces the amount of moisture on the surfaces [16]. Copper alloy surfaces and silver ion surfaces have been reported to have greater antibacterial activity under the optimum conditions required by ISO 22196, compared to real environmental conditions [36,52]. Varghese et al. [26] performed ISO 22196 at both room temperature (20–25 °C) and the temperature required by the standard (35 °C) and concluded that inflated values of antibacterial activity were obtained at 35 °C. Another problem that was reported was difficulty in maintaining the viability of the inocula on the controls after only 6 h of incubation of the test specimens. Considering this issue, some studies have been performed at an incubation temperature of test specimens of between 20 and 25 °C, which is lower than that recommended in ISO 22196 [11], in order to deter the possible inflation of antimicrobial activity and a decrease in the viability of the inoculum [17,33,41]. Michels et al. [73], who used the Japanese Industrial Standard, JIS Z 2801, on which ISO 22196 is based, reported that the antibacterial activity of silver-ion-containing materials is profoundly affected by both temperature and relative humidity. The authors demonstrated that this material showed antibacterial activity only at a high temperature (35 °C) and high relative humidity (>90%), while no significant antibacterial efficacy was observed at 35 and 20% relative humidity and, more importantly, at 20 °C and 24% relative humidity. This finding suggests that assessing the efficacy of antimicrobial materials intended for everyday scenarios in hospital, household and industry settings cannot be carried out using the standard protocol as described. Inflated results may enable false claims of antibacterial efficacy, perpetuating the lack of confidence in these materials and misleading institutions into believing that efforts are being made to complement existing cleaning and disinfection protocols.

### 3.3. Contact Time

Another factor that may play a major role in the efficacy of an antibacterial coating is the contact time of the inocula with the treated surface during the incubation of test specimens. A total of 10 authors reported changes in contact time or additional time points [24,25,26,30,41,42,52,60,68]. Ashara et al. [60] studied antimicrobial activity for 7, 14, 21 and 28 days instead of the 24-h exposure specified in the standard. Barzan et al. [52] reported that in order to better understand the kinetics of killing efficacy and to emphasise the primary variations in the size and surface coverage of the AgNPs, the contact time of the inocula with the treated surface was reduced to 5 h instead of 24 h. Additional time points were assessed by Bento de Carvalho et al. [68] (1, 10 and 20 min), Różańska et al. [41] (0, 60, 120, 180, 240 and 300 min), Torlak et al. [24] (1 and 6 h), Tramuta et al., [37] (1, 3 and 6 h) and Varghese et al. [25,26] (0, 1, 4, 6 h). Bazant et al. [30,42] reported some modifications to reduce the risk of false results and avoid incorrect overestimating of antibacterial activity caused by the slower growth rate. After inoculation, contact time was assessed at 24 h, as required, and after 48 h at 35 °C.

### 3.4. Surface Topography

Another critical element is the antibacterial surface’s topography. While highly smooth (polished) surfaces do not favour bacterial adherence and biofilm formation, rough (unpolished) surfaces have surface imperfections that favour it. In addition to these readily apparent characteristics, the design of the surface is important because bacteria preferentially attach to and colonise porous surfaces rather than dense materials. The contact surface that is available for adhesion and, thus, the binding potential, is increased by microscopic scratches or grooves that are roughly the same size as the bacteria. However, this binding is weaker if these flaws are significantly larger or smaller than the size of the bacteria [74]. Environmental conditions such as pH, temperature, surface hydrophobicity or hydrophilicity and surface topography are also important factors affecting bacterial adhesion [75]. Surface topography can play a decisive role in the antibacterial activity of a compound. Jana et al. [76] have reported that zinc additives can alter the roughness of surfaces, thus impacting their antibacterial potential. Surface modifications that may change its topography due to subsequent fouling or wear are important to characterise, to either achieve or maintain the antibacterial effect of the compound that is used. Surface wettability is also important; while hydrophobic surfaces allow a slower drying time due to the formation of droplets on the surface, hydrophilic surfaces allow an even spread of the liquid and a faster drying time for the same volume [10,16].

ISO standards are the reference methods for food microbiological regulations and are widely used for food microbiological analysis, aiming to regulate and reduce duplication, minimise errors and speed up the time to market. Standard protocols are needed and are crucial to ensure the reproducibility of the procedure and acceptance by accredited laboratories. Any adjustments made to the method must be documented in the final test report, as both major and minor modifications can affect the results. In the case of ISO 22196, the modifications are mainly applied since incubation of the inoculated and treated surfaces at 35 °C and at >90% relative humidity, as required by the standard, will lead to inflated antibacterial activity, this fact being the main issue of the validity of this standard. To better discern if the antibacterial activity of a coating is real or merely efficient due to optimum environmental conditions, real-life settings (i.e., a room temperature of 20 to 25 °C and 40–50% relative humidity) should be included in the standard to avoid this drawback. ISO 22196 [11] is a good and reliable Tier 1 test to determine the antimicrobial activity of treated surfaces under artificial conditions, while Tier 2 testing should also be employed to emulate real-life conditions and use and to evaluate product characteristics such as durability and the maintenance of total antimicrobial activity with wear [77,78]. Knobloch et al. [36] and Ojeil et al. [18] have proposed alternative methodologies to assess the antibacterial activity of AMCs.

## 4. Conclusions

Through a closer look into studies using ISO 22196, this review has highlighted both the importance and limitations of the protocol [11] as a standardised method for assessing the activity of antimicrobial coatings on surfaces. Although this method is widely used to evaluate the antimicrobial properties of treated plastics and non-porous surfaces, its artificial test conditions pose challenges in accurately reflecting real-life scenarios in healthcare, domestic and industrial settings. The simplicity, affordability and wide availability of the protocol make ISO 22196 [11] an essential Tier 1 test for the initial screening of antimicrobial coatings. However, its restrictive conditions, including temperature, humidity and inoculum concentrations, have shortcomings when it comes to accurately reflecting practical environments. Consequently, Tier 2 tests, simulating real-life environments, are crucial to validate and understand the true efficacy of antimicrobial coatings in practical usage. The modifications introduced in various studies attempt to bridge this gap by simulating real conditions, but they often lack standardisation and consistency between studies, affecting the reliability and comparability of the results. Given that several factors influence the validity of the ISO 22196 standard, including inoculum density, temperature, humidity, contact time and surface topography, when these variables are altered, they significantly affect the reported antimicrobial efficacy, necessitating a cautious approach to interpreting the results.

It should be emphasised that while the ISO 22196 standard remains an invaluable standardised method for assessing surfaces coated with antimicrobials, its limitations in representing real-world conditions require further research and modification. The standardisation of testing protocols, the transparent communication of modifications and a concerted effort to develop stepwise testing strategies are essential to ensure the reliability, reproducibility and practical relevance of assessing the effectiveness of antimicrobial surface coatings for healthcare, domestic and industrial applications.

## Figures and Tables

**Table 1:** Literature on the different reported results of studies using the ISO 22196 standard.

Application	Bacterial Strains	ISO 22196 Modification	Detailed Modification Protocol	Result Reporting	Reference
Biomaterials (Resin-based bone cement)	*Escherichia coli* NBRC 3972 *Staphylococcus aureus* NBRC 12732	Culture medium	1/500 Nutrient broth substituted with rich media (Mueller–Hinton broth and fetal bovine serum)	Number of viable bacteria CFU	[22]
Healthcare settings (HVAC aluminium ducts)	*Legionella pneumophila* ATCC 33152 *S. aureus* ATCC 6538 *Pseudomonas aeruginosa* ATCC 15422 *E. coli* ATCC 8739 *Candida albicans* ATCC 10231 *Aspergillus niger* ATCC6275)	Culture medium	Tryptic soy agar was used to determine viable bacteria instead of plate count agar	Germicidal effect (ULOG_10_)	[23]
Food packaging (Polypropylene)	*Bacillus cereus* ATCC 11778 *Listeria monocytogenes* ATCC 7644 *S. aureus* ATCC 25923 *Cronobacter sakazakii* ATCC 51329 *Salmonella* Typhimurium ATCC 14028 *E. coli* O157:H7 NCIMB 13861	Incubation time and bacterial suspension volume	The volume of the inoculum was reduced to 200 μL from 400 μL, using the test specimen area indicated by the standard Additional incubation times of 1 h and 6 h	Log CFU/sample	[24]
Healthcare settings (Copper surfaces)	*S. aureus* NCIMB 9518	Incubation humidity and bacterial recovery method	A saturated solution of zinc sulphate was used to maintain high humidity Recovery of bacterial cells was performed on stomacher bags and not on the Petri dishes used for incubation Maximum recovery diluent used for bacterial inocula preparation instead of nutrient broth	Log_10_ CFU/cm^2^	[18]
Healthcare settings (Borosilicate glass)	*S. aureus* ATCC 6538 *E. coli* ATCC 8739 *Enterococcus faecalis* NCIMB 775 *P. aeruginosa* NCIMB 10421	Incubation temperature and different time points; cover film	The incubation temperature of the test specimen was set at between 20 and 25 °C instead of 35 °C Additional time points were included: 1 h, 2 h, 4 h and 6 h Glass covers were used instead of plastic film for covering the inoculum during incubation	Log reduction factor	[25]
Healthcare settings (Borosilicate glass)	*E. coli* ATCC 8739 *S. aureus* ATCC 6538 *P. aeruginosa* 10421 *Acinetobacter baumannii* *Klebsiella pneumoniae* *E. coli * EMRSA15 MRSA 1599 MRSA 1665 MRSA NCTC10492 *Stenotrophomonas maltophilia* *Enterococcus faecium* (VRE)	Incubation temperature and different time points; cover film	The incubation temperature of the test specimen was set at between 20 and 25 °C instead of 35 °C Additional time points were included: 1 h, 2 h, 4 h and 6 h Glass covers were used instead of plastic film for covering the inoculum during incubation	Log viable count CFU	[26]
Medical devices (Orthopaedic implants)	*E. coli* ATCC 29522 *S. aureus* ATCC 6538	Bacterial suspension volume; neutraliser choice; culture medium	The volume of the inoculum was reduced to 300 μL from 400 μL to be proportional to the test specimen and the sterile cover-film area Phosphate-buffered saline was used instead of casein peptone lecithin polysorbate broth MacConkey agar was used in place of plate count agar for bacterial recovery counts	Percent bacterial death (%)	[27]
Medical devices (PVC)	*E. coli* ATCC 8739 *S. aureus* ATCC 6538P	No modifications reported	No modifications reported	Antibacterial activity (R)	[28]
Various environmental sites (Stainless steel and glass)	*E. coli* 72002	Culture medium	Lysogeny broth was used for bacterial inocula instead of 1/500 nutrient broth and nutrient agar was used in place of plate count agar for bacterial recovery counts	Logarithmic reduction of bacterial load	[29]
Medical plastics (PVC)	*E. coli* ATCC 8739 *S. aureus* ATCC 6538P	Incubation time	48-hour incubation of the test specimens instead of 24 h to reduce the risk of false results	Antibacterial activity (R)	[30]
Medical devices (Epoxy resin-based sealers)	*Streptococcus oralis* DSM 20627	Bacterial suspension volume; bacterial recovery method	The volume of the inoculum was reduced to 200 μL from 400 μL to be proportional to the test specimen and the sterile cover film area Bacterial recovery performed with less neutraliser volume	Data not shown	[31]
Medical devices and various environmental sites (Polymer film)	*S. aureus* MRSA	Bacterial suspension volume and incubation method	The volume of the inoculum was reduced to 200 μL from 400 μL to be proportional to the test specimen and the sterile cover film area Test specimens incubated with 5% CO_2_	Antibacterial activity (R)	[32]
Clinical use (Film surfaces)	*E. coli* 9927 *K. pneumoniae* 9936 *S. aureus* 95 *S. aureus* 175	Incubation temperature and culture medium	Test specimens were incubated at room temperature instead of at 35 °C Mueller–Hinton broth was used for bacterial inocula growth rather than 1/500 nutrient broth and Mueller–Hinton agar was used to determine viable bacteria instead of plate count agar	CFU/mL	[17]
Food packaging (Biodegradable multilayer systems)	Feline calicivirus F9 Murine norovirus MNV-1	Modified to virucidal activity	Specimen size of 3 × 3 cm and cover film size of 2.5 × 2.5 cm instead of 5 × 5 cm and 4 × 4 cm, respectively	Reduction	[33]
Medical devices (Zirconia)	*S. aureus* NBRC122135 *Streptococcus mutans* MT8148 *E. coli* NBRC3972 *Aggregatibacter actinomycetemcomitans* ATCC33384	Bacterial suspension concentration	Bacterial suspension concentration higher than stated on the standard (0.4 to 3.0 × 10^8^ CFU/mL)	Log viable cells (CFU)	[34]
Healthcare settings (Ceramic tiles)	*S. aureus* ATCC 3359	Different culture medium and diluent	Columbia sheep blood agar was used to determine viable bacteria instead of plate count agar Tryptic soy broth was chosen instead of casein peptone lecithin polysorbate broth	Antibacterial activity (R)	[35]
Medical devices (Silicone elastomer)	*S. aureus* ATCC 25923 *E. coli* ATCC 8739 *E. faecalis* ATCC 29212 *A. baumannii* ATCC 19606 *P. aeruginosa* ATCC 25375 *K. pneumoniae* DSM 16609 *Staphylococcus epidermidis* DSM 18857 *Enterobacter cloacae* DSM 30054	Bacterial suspension volume	The volume of the inoculum was reduced to 200 μL from 400 μL to be proportional to the test specimen and the sterile cover film area	Antibacterial activity (R)	[36]
Veterinary clinical devices (Honey-based membranes)	*E. coli* *Proteus mirabilis* *P. aeruginosa*	Different incubation times and bacterial suspension	Bacterial suspension was performed on undiluted nutrient broth Additional incubation times of 1 h, 3 h and 6 h	Log CFU/sample	[37]
Bioengineering Applications (non-specified)	*S. aureus* *E. coli* *C. albicans*	Incubation and recovery method and culture medium	The volume of the inoculum was reduced to 150 μL from 400 μL to be proportional to the test specimen and the sterile cover film area Test specimens incubated in 48-well plates To recover bacterial cells from the surface, an additional step of sonication was added during the recovery of bacteria from the test specimen Tryptic soy agar was used to determine viable bacteria instead of plate count agar	Loss of viability (%)	[38]
Medical devices (Urinary catheters)	*E. coli* ATCC 8739	Bacterial suspension volume; recovery method	The volume of the inoculum was reduced to 200 μL from 400 μL to be proportional to the test specimen and the sterile cover film area To recover bacterial cells from the surface, an additional step of sonication was added during the recovery of bacteria from the test specimen	Log CFU/mL	[39]
Healthcare settings (Nanotubes)	*Listeria innocua* *L. monocytogenes* *E. coli* *S. aureus*	Bacterial suspension volume; incubation method	The volume of the inoculum was reduced to 1000 μL from 400 μL to be proportional to the test specimen and the sterile cover film area Incubation temperature of the test specimen of 4 °C; further treatment (LED lamp exposure) during incubation Agar was poured directly into the test specimen for bacterial recovery	CFU	[40]
Healthcare settings (Metal samples)	*A. baumannii* *Acinetobacter pittii* *Acinetobacter lwoffii*	Bacterial suspension volume; incubation temperature; incubation time and bacterial recovery method	The volume of the inoculum was reduced to 100 μL from 400 μL to be proportional to the test specimen and the sterile cover film area Additional time points were studied: 60, 120, 240 and 300 min Incubation of the test specimens was carried out at 22 °C	CFU/mL	[41]
Plastic medical devices Sanitary, hygienic or other interior applications (Propylene-based elastomer)	*E. coli* ATCC 8739 *S. aureus* ATCC 6538P	Incubation time	48-hour incubation of the test specimens instead of 24 h to reduce the risk of false results	Antibacterial activity (R) and efficiency (%)	[42]
Food contact materials (Food-grade polymeric matrices)	*S. aureus* CNRZ3	Bacterial suspension volume; choice of neutraliser	The volume of the inoculum was reduced to 200 μL from 400 μL to be proportional to the test specimen and the sterile cover film area Dey–Engley neutraliser was chosen instead of casein peptone lecithin polysorbate broth	R: Log_10_ CFU/cm^2^	[2]
Medical devices (Orthodontic cement)	*S. aureus* 6538	Culture medium	Tryptic soy agar was used to determine viable bacteria instead of plate count agar	Log reduction compared to control	[43]
Food packaging (Polyethylene and polypropylene)	*S. aureus* *E. coli*	Modifications reported	No detailed modification protocol	Antimicrobial efficacy (%)	[44]
Medical devices (Titanium alloy)	*S. aureus* ATCC 29214 *E. coli* ATCC 25922	No modifications reported	No modifications reported	Number of Viable Bacteria CFUs	[45]
Bioactive materials (Glass)	*S. aureus* 43300	Bacterial suspension volume; incubation of test specimens	The volume of the inoculum was reduced to 100 μL from 400 μL to be proportional to the test specimen and the sterile cover film area Test specimens incubated in 12 multi-well plates	CFU count (Log_10_)	[46]
Medical devices (Glass)	*E. coli* ATCC 11229 *P. aeruginosa* ATCC 9027 *L. monocytogenes* ATCC 19114 *S. aureus* ATCC 6538 *C. albicans* ATCC 10231	Bacterial recovery method; incubation temperature and bacterial recovery incubation time	The volume of the inoculum was increased to 500 μL from 400 μL to be proportional to the test specimen and the sterile cover film area Test specimens were incubated in tubes instead of Petri dishes Incubation of test specimens was carried out at 30 °C instead of the standard recommended 37 °C Bacterial recovery incubation time was reduced to 24 h instead of 48 h	Log CFU/cm^2^	[47]
Food packaging (Polyethylene terephthalate and aluminium film)	*E. coli* DSM 1576 *S. aureus* DSM 346	No modifications reported	No modifications reported	CFU/film	[48]
Healthcare settings (Paint samples)	*E. coli* ATCC 25922 *Klebsiella variicola* ATCC 31488 *S. aureus* ATCC 25923 *B. cereus* *E. faecalis* NCTC 775	Neutraliser choice	TSB neutralising solution was used instead of casein peptone lecithin polysorbate broth	Antibacterial activity (R)	[49]
Medical devices (Ethylene vinyl acetate surface)	*S. aureus* *Streptococcus sobrinus* OMZ176	Bacterial suspension volume and culture medium	Bacterial suspension concentration lower than that recommended, at 1.0 × 10^4^ CFU/mL Incubation performed on 6-well plates Brain–heart infusion medium used for bacterial inocula instead of nutrient broth	Log CFUs	[50]
Medical devices (Commercially pure titanium and austenitic steel)	*S. aureus*	Bacterial recovery; culture medium	To recover bacterial cells from the surface, an additional step of sonication was added during the recovery of bacteria from the test specimen Columbia sheep blood agar was used to determine viable bacteria instead of plate count agar	Log CFU/ biomaterial	[51]
Medical devices (Biomaterial)	*E. coli* ATCC 8739	Bacterial suspension volume, concentration and incubation time	The volume of the inoculum was reduced to 100 μL from 400 μL to be proportional to the test specimen and the sterile cover film area 1 × 10^6^ CFU/mL of bacteria were inoculated instead of the target concentration range of 2.5 × 10^5^–10 × 10^5^ CFU/mL Incubation of the test specimens was reduced from 24 h to 5 h	Antibacterial activity (R)	[52]
Biocomposite Material (Polylactide biocomposite)	*S. aureus* ATCC 6538P *E. coli* ATCC 8739	No modification reported	No modification reported	Antibacterial activity (R)	[53]
Healthcare settings (Glass surface)	*S. aureus* DSM 346 MRSA DSM 11729	Culture medium; bacterial suspension volume	The volume of the inoculum was reduced to 100 μL from 400 μL to be proportional to the test specimen and the sterile cover film area TSB was used as the diluent during the neutraliser phase	CFU	[54]
Agricultural and horticultural applications (Plastic samples)	*Agrobacterium tumefaciens* *Xanthomonas campestres* *Pseudomonas corrugata * *Pseudomonas brassicacearum* *Pseudomonas syringae*	Bacterial inoculum concentration	It is reported that a concentration of 10^6^ was used rather that the target of 6 × 10^5^ CFU/mL	Antibacterial activity (R)	[55]
Food packaging (Polymeric Surface)	*S. aureus* CCM 4516 *E. coli* CCM 4517	Bacterial suspension volume	The volume of the inoculum was reduced to 100 μL from 400 μL to be proportional to the test specimen and the sterile cover film area	Antibacterial activity (R)	[33]
Water devices (Polyethylene surface)	*E. coli* ATCC 15597 *E. faecalis* ATCC 29212	Bacterial suspension volume, diluent and growth medium	Luria–Bertani broth instead of Nutrient broth for inoculum preparation The volume of the inoculum was reduced to 200 μL from 400 μL to be proportional to the test specimen and the sterile cover film area Water was used as diluent in place of casein peptone lecithin polysorbate broth	Colony counts (CFU/sample)	[56]
Medical devices (Silicone and polyurethane surfaces)	*S. aureus* ATCC 6538 *E. coli* ATCC 8739 *S. epidermidis* ATCC 43862 *Serratia marcescens* ATCC 35984 *K. pneumoniae* ATCC 13883 *E. faecalis* ATCC 47077	Additional incubation temperatures	Test specimens were incubated at 4 °C, 22 °C and 35 °C	Log reduction and Reduction (%)	[57]
Food industry (Stainless-steel surfaces)	*E. coli* NBRC3972 *S. aureus* NBRC12732	Different time points and recovery diluent/neutraliser	8-hour incubation period instead of the stipulated 24 h Saline water was used for the recovery of bacterial cells instead of casein peptone lecithin polysorbate broth	Antibacterial activity (R) and Bacterial count sample/ cm^2^	[58]
Comercial paint (Paint)	*E. coli* (ATCC 25922) *S. aureus* (ATCC 29213) *E. faecalis* (ATCC 29212) *Bacillus subtilis* *Bacillus pumilus* *Bacillus altitudinis*	Bacterial suspension volume; culture medium	The volume of the inoculum was reduced to 200 μL from 400 μL using the test specimen area indicated by the standard Mueller–Hinton agar was used to determine viable bacteria instead of plate count agar	Antibacterial activity (R)	[59]
Biomaterial (Tissue conditioner)	*C. albicans* ATCC 48130 *S. aureus* ATCC 6538P	Incubation time	7-, 14-, 21- and 28-day incubation instead of 24-hour incubation	Viable cells (CFU)	[60]
Various environmental sites (Paint samples)	*E. coli* ATCC 25922 *K. variicola* ATCC 31488 *S. aureus* ATCC 25923 *Bacillus cereus * *E. faecalis* NCTC 775	Minor modifications reported	No detailed modification protocol	Antibacterial activity (R)	[61]
Food packaging (Plastic film)	*S. aureus* ATCC 25923	Modifications reported	No detailed modification protocol	Log CFU/cm^2^	[62]
Packaging industry (Polymeric material)	*E. coli* ATCC 8739 *S. aureus* ATCC 6538P *P. aeruginosa* ATCC 13388 *A. tumefaciens* *X. campestres* *P. corrugata* *P. brassicacearum* *P. syringae*	No modifications reported	No modifications reported	Antibacterial activity (R) and % Reduction	[63]
Medical devices (Titanium plates)	*S. aureus* ATCC 25923 *E. coli* ATCC 25922	No modifications reported	No modifications reported	Antimicrobial activity (%)	[64]
Healthcare and community settings (Plastic surfaces)	*S. aureus* CIP 4.83 *E. coli* CIP 53.126	Culture medium	Trypticase soy agar was used to determine viable bacteria instead of plate count agar	Log Reduction	[65]
Non-Woven Fabrics (Face masks)	*S. aureus* ATCC 6538 *E. coli* CECT 434	Test specimen size	Specimen size of 3 × 3 cm and cover film size of 2 × 2 cm instead of 5 × 5 cm and 4 × 4 cm, respectively	Antimicrobial activity (R)	[66]
Healthcare settings (Enamel)	*S. aureus* ATCC 6538 *E. coli* ATCC 8739 *C. albicans*	Surface sterilisation, inoculated surfaces incubation and bacterial recovery	Additional UV light sterilisation treatment Phosphate-buffered saline was used instead of casein peptone lecithin polysorbate broth An additional step was included, using rotation to dissociate the bacteria from the surfaces	Recovered bacteria (%)	[67]
Domestic kitchens (PVC, glass and stainless steel surfaces)	*E. coli* ATCC 25922 *L. monocytogenes * Scott A *A. baumannii* ESB260	Bacterial suspension volume; incubation temperature; choice of neutraliser	The volume of the inoculum was reduced to 200 μL from 400 μL to be proportional to the test specimen and the sterile cover film area The incubation temperature was lowered to 22 °C from 35°C Dey–Engley neutraliser was chosen instead of casein peptone lecithin polysorbate broth	CFU/cm^2^	[68]

ATCC—American Type Culture Collection; CCM—Czech Collection of Microorganisms, Masaryk University, Brno, Czech Republic; CFU—colony-forming units; CIP—Collection of Institut Pasteur, Biological Resource Center of Institut Pasteur (CRBIP), Paris, France; CNRZ—Centre National de Recherches Zootechniques, Jouy-en-Josas, France; DSM—DSMZ-Deutsche Sammlung von Mikroorganismen und Zellkulturen GmbH, Braunschweig, Germany; ESB—Escola Superior de Biotecnologia, Porto, Portugal; MRSA—methicillin-resistant *Staphylococcus aureus*; NBRC—NITE Biological Resource Center, Department of Biotechnology, National Institute of Technology and Evaluation, Kisarazu, Chiba, Japan; NCIMB—National Collection of Industrial, Food and Marine Bacteria, NCIMB Ltd., Aberdeen, Scotland, UK; NCTC—National Collection of Type Cultures, Central Public Laboratory Service, London, UK; R—antibacterial activity; VRE—vancomycin-resistant enterococci.

## Data Availability

Not applicable.
